# Aging- and alcohol-associated spatial transcriptomic signature in mouse acute pancreatitis reveals heterogeneity of inflammation and potential pathogenic factors

**DOI:** 10.1007/s00109-024-02460-6

**Published:** 2024-06-28

**Authors:** Rachel R. Tindall, Yuntao Yang, Isabella Hernandez, Amy Qin, Jiajing Li, Yinjie Zhang, Thomas H. Gomez, Mamoun Younes, Qiang Shen, Jennifer M. Bailey-Lundberg, Zhongming Zhao, Daniel Kraushaar, Patricia Castro, Yanna Cao, W. Jim Zheng, Tien C. Ko

**Affiliations:** 1 Department of Surgery, UTHealth at Houston, Houston, TX 77030 USA; 2 McWilliams School of Biomedical Informatics, UTHealth at Houston, Houston, TX 77030 USA; 3 Center for Laboratory Animal Medicine and Care, UTHealth at Houston, Houston, TX 77030 USA; 4 Department of Pathology, George Washington University School of Medicine and Health Sciences, Washington, DC 20037 USA; 5 Department of Interdisciplinary Oncology, Louisiana State Univ. Health Sciences Center, New Orleans, LA 70112 USA; 6 Department of Anesthesiology, Critical Care and Pain Medicine, UTHealth at Houston, Houston, TX 77030 USA; 7 Center for Precision Health, McWilliams School of Biomedical Informatics, UTHealth at Houston, Houston, TX 77030 USA; 8 Genomic and RNA Profiling Core, Baylor College of Medicine, Houston, TX 77030 USA; 9 Human Tissue Acquisition & Pathology Core, Baylor College of Medicine, Houston, TX 77030 USA

**Keywords:** Aging, Alcohol, Acute pancreatitis, Alcohol-associated acute pancreatitis mouse model, Visium spatial transcriptomics, Differentially expressed genes

## Abstract

**Abstract:**

The rapidly aging population is consuming more alcohol, leading to increased alcohol-associated acute pancreatitis (AAP) with high mortality. However, the mechanisms remain undefined, and currently there are no effective therapies available. This study aims to elucidate aging- and alcohol-associated spatial transcriptomic signature by establishing an aging AAP mouse model and applying Visium spatial transcriptomics for understanding of the mechanisms in the context of the pancreatic tissue. Upon alcohol diet feeding and caerulein treatment, aging mice (18 months) developed significantly more severe AAP with 5.0-fold increase of injury score and 2.4-fold increase of amylase compared to young mice (3 months). Via Visium spatial transcriptomics, eight distinct tissue clusters were revealed from aggregated transcriptomes of aging and young AAP mice: five acinar, two stromal, and one islet, which were then merged into three clusters: acinar, stromal, and islet for the comparative analysis. Compared to young AAP mice, > 1300 differentially expressed genes (DEGs) and approximately 3000 differentially regulated pathways were identified in aging AAP mice. The top five DEGs upregulated in aging AAP mice include *Mmp8*, *Ppbp*, *Serpina3m*, *Cxcl13*, *and Hamp* with heterogeneous distributions among the clusters. Taken together, this study demonstrates spatial heterogeneity of inflammatory processes in aging AAP mice, offering novel insights into the mechanisms and potential drivers for AAP development.

**Key messages:**

- Mechanisms regarding high mortality of AAP in aging remain undefined.
- An aging AAP mouse model was developed recapturing clinical exhibition in humans.
- Spatial transcriptomics identified contrasted DEGs in aging vs. young AAP mice. Top five DEGs were *Mmp8*, *Ppbp*, *Serpina3m*, *Cxcl13*, and *Hamp* in aging vs. young AAP mice.
- Our findings shed insights for identification of molecular drivers in aging AAP.

**Supplementary Information:**

The online version contains supplementary material available at 10.1007/s00109-024-02460-6.

## Introduction

Our society is rapidly aging. As of 2015, individuals aged ≥ 65 constituted 9% of the global population, which is projected to be 12% by 2030 and 17% by 2050 [1]. Alcohol use is steadily increasing in the aging population. In 2020, 12.1% of women and 21.9% of men reported alcohol consumption, a significant increase of 6.5% and 7.6%, respectively, from 1990 [2]. Alcohol consumption is a known risk factor for a myriad of diseases [3], and elderly adults are especially susceptible to the adverse impact of alcohol, contributing to an even greater risk of diseases such as acute pancreatitis (AP) [4].

AP accounts for 300,000 yearly visits to the emergency department (ED) [5, 6]. One in five AP patients progresses to severe disease resulting in 20% mortality [5]. Age ≥ 65 is associated with worse outcomes and is a strong predictor for hospital admission from the ED [7, 8]. Alcohol is a common cause of AP, second only to gallstone-induced AP [9], and is associated with worse outcomes [10]. AP will continue to be a major source of healthcare expenditure and mortality in the aging population [11]. Thus, elucidating the molecular mechanisms involved in age- and alcohol-associated AP is imperative to developing targeted therapeutics and improving outcomes.

The pancreas has distinct tissue types characterized by exocrine, endocrine, and stromal functions. Exocrine tissue is composed of acinar cells, which are the primary injured cell type in AP causing pancreatic autodigestion and inflammation [12]. Due to the heterogeneous nature of pancreatic tissue, AP affects all pancreatic cell types, and the intercellular dynamics of AP cannot be accurately studied using traditional bulk technologies. Single-cell technologies have allowed examination of individual cellular phenotypes [13–16]. However, tissue dissociation for single-cell isolation leads to loss of spatial organization and possible gene alteration [17, 18]. Recently developed spatial transcriptomics technology maintains the original spatial orientation of tissue samples and preserves the gene expression in its true tissue context enabling the exploration of the function in relation to morphology [17–21]. This technology has been used to investigate pancreatic development and cancer [22, 23].

In this study, we established a unique aging-associated AAP mouse model, employed Visium spatial transcriptomics to the pancreatic tissue sections, and mapped the whole transcriptome to study the gene expression profiles. To our knowledge, this is the first application of spatial transcriptomics in AP, providing new insights into the localized genetic expression profile of the heterogeneously inflamed pancreatic tissue. Compared to young mice, aging mice developed more severe AAP, which was associated with differentially expressed genes (DEGs) and pathways related to inflammation. These findings shed light on the molecular mechanisms of AAP in aging mice, providing direction for future studies.

## Materials and methods

### Animals and AAP model

Young (3 months old (m)) and aging (18 m) C57BL/6 male mice were purchased from the Jackson Laboratory (Bay Harbor, ME) and housed in a 23 °C ambient temperature, 12:12 light-dark cycle facility. Mice were acclimated to Lieber-DeCarli control liquid diet (CON, #F1340SP, Bio-Serv, Flemington, NJ) for 1 week and randomly assigned to experimental groups. Mice were fed CON diet (# F1340SP), or alcohol diet (EtOH, # F1341SP) with incremental increases from 2 to 5% EtOH in 1 week and then stay on 5% EtOH for 2 weeks. Mice were given water ad libitum. Mice received caerulein (CAE, from Bachem Americas, Inc, Torrance, CA) at 50 µg/kg, 3 hourly intraperitoneal injections at the end of 2 weeks’ feeding, or PBS as CAE control (*n* = 4**–**6 mice/group). Mice were euthanized 16 h after the last injection. The blood and pancreas were harvested. The pancreata were fixed in 10% formalin and embedded in paraffin (FFPE).

### Morphologic examination

FFPE pancreas samples were sectioned and stained with hematoxylin and eosin (H&E). AP lesions were evaluated as described previously [24–26].

### Biochemical assays

Serum alcohol concentration was measured using an Ethanol Assay Kit (Abcam, Waltham, MA) as described by the manufacturer. Serum amylase levels were determined using the Phadebas Amylase Test (Magle Life Sciences, Cambridge, MA) as previously described [25].

### Spatial transcriptomics

Visium spatial transcriptomics was performed on FFPE as recently described [27]. FFPE tissues were sectioned at 10 µm thickness onto a 10 × Genomics Spatial Gene Expression slide (10 × Genomics, San Francisco, CA), and 6.5 mm^2^ sections were placed on capture areas. Slides were fixed, stained with H&E, and imaged. Sample quality checks were conducted with isolated RNA from FFPE sections using the NanoDrop Agilent Bioanalyzer. The Visium Spatial for FFPE v1 Gene Expression Kit was used for library preparation per manufacturer’s instructions. For sequencing, 150 pM of the equimolarly pooled library was loaded onto the NovaSeq 6000 S4 flowcell and sequenced at the recommended 28-10-10-50 read configuration. PhiX Control v3 adapter-ligated library (Illumina p/n FC-110-3001) was spiked-in at 2% by weight to ensure balanced diversity and to monitor clustering and sequencing performance. A minimum of 300 million read pairs per sample was sequenced. The raw FASTQ files were generated using the *spaceranger mkfastq* function in the 10 × Genomics Space Ranger.

### Computational methods for data analysis

#### Visium spatial raw data processing

Manual image preprocessing was performed using 10 × Genomics Loupe Browser 6.4.0 to remove spleens, air bubbles, and debris. The *spaceranger* count function in 10 × Genomics Space Ranger 1.3.1 was utilized to align the raw FASTQ sequences to the mouse reference genome mm10, remove non-tissue-associated barcodes and non-targeted genes, and count barcodes and unique molecular identifiers (UMIs).

#### Normalization, data integration, clustering, and dimensionality reduction

The filtered feature-barcode matrix and its corresponding spatial image were loaded and analyzed using functions from the R package Seurat 4.3.0 [28]. The *SCTransform* function was utilized to normalize gene expression values for each sample, and highly variable genes (*n* = 3000) were selected for data integration. To perform the comparative analysis between aging and young AAP mice and correct batch effect, samples were integrated using the *FindIntegrationAnchors* function and the *IntegrateData* function. Principal component analysis (PCA) was performed using the *RunPCA* function, and 30 correlated components were selected for identifying different tissue clusters using the graph-based clustering algorithm (resolution = 0.3) and conducting dimensionality reduction for visualizing clusters using tools of the uniform manifold approximation and projection (UMAP) [29] and t-distributed stochastic neighbor embedding (t-SNE) [30]. To determine the tissue type for each cluster, marker genes were extracted using the Wilcoxon Rank Sum test, applied within the *FindAllMarkers* function.

#### Identification of cell type via single-cell (sc)RNA-seq data integration

The spatial transcriptomics data were integrated with scRNA-seq data from normal pancreas sample (GSM2906458) in the GSE108097 dataset [31]. To map cell-type annotations from the scRNA-seq dataset to the spatial transcriptomics dataset, the *FindTransferAnchors* and *TransferData* functions in Seurat were used to generate a prediction score for each spatial spot and cell type. The major cell type for each spatial spot was selected based on the highest prediction score.

#### DEG analysis

DEG analysis started with the merging of tissue clusters representing identical tissue types into a single acinar, stromal and islet cluster. For each tissue cluster, DEGs comparing Aging_AAP clusters with Young_AAP clusters were identified through the *FindMarkers* function. DEGs were filtered using criteria of false discovery rate (FDR) < 0.05, log2FC (fold changes of gene expression) > 0.25 (for upregulation) or < -0.25 (for downregulation), and percentage of gene expression criteria of pct.1 > 0.1 and pct.2 > 0.1.

#### Differential pathway analysis

The Over-Representation Analysis (ORA) of Gene Ontology (GO) was applied to the filtered DEGs using the *enrichGO* function from the R package clusterProfiler 4.6.0 [32]. Differentially regulated pathways were filtered using criteria of FDR < 0.05.

### Quantitative PCR

Quantitative (q)PCR was conducted as previously described [33, 34] using specific gene probes for mouse *Interleukin (Il)6* (Mm00446190_m1), *Il10* (Mm01288386_m1), *Ptgs2* (Mm00478374_m1), *Hamp* (Mm04231240_s1), and 18S (Hs99999901_s1) from ThermoFisher Scientific (Waltham, MA, USA). Specific signals were normalized to 18S signals.

### Statistics

Data are expressed as mean ± standard error (SEM). Statistical significance was determined using t-test for two group comparison and ANOVA for multiple group comparison. *P* values < 0.05 were considered significant. All statistics were performed using GraphPad Prism version 9 (La Jolla, CA USA) except Visium spatial transcriptomic data analysis described earlier.

## Results

### Aging mice had greater inflammatory responses in AAP

**Fig. 1:**
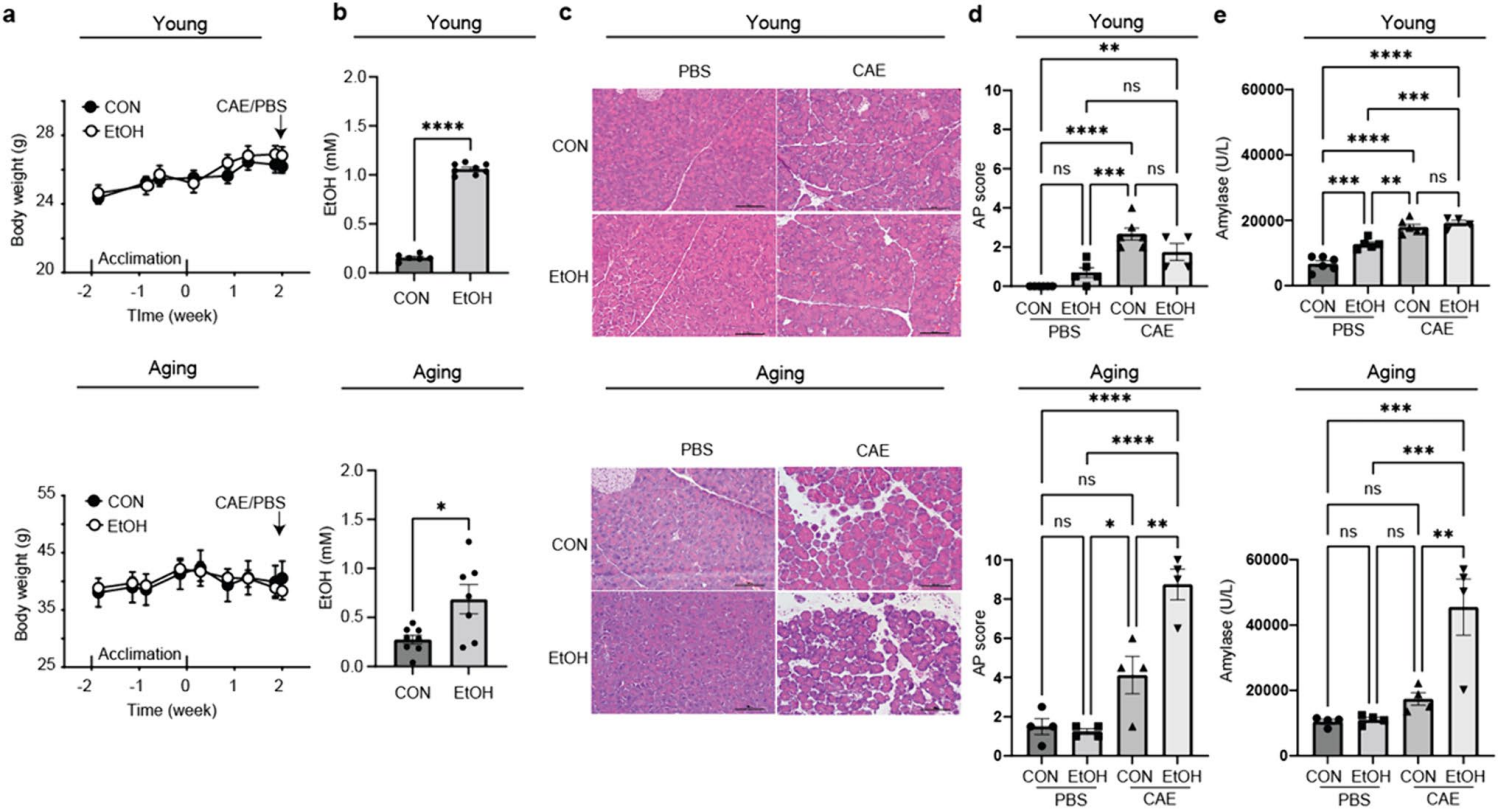
Alcohol feeding sensitizes aging mice to a low-dose caerulein-induced acute pancreatitis. Mice were acclimated to Lieber-DeCarli CON diet and then fed incrementally increased EtOH from 2, 4, and 5% for 1 week and remained on 5% for 2 weeks, and the control mice were fed CON diet. CAE was injected (50 µg/kg, 3 hourly injections, ip) at the end of two week, and PBS injections served as the control of CAE. The mice were euthanized 16 h after CAE injections; the blood and pancreata were harvested. **a** Body weight. **b** Serum EtOH level at the experiment endpoint. **c** H&E images of the pancreatic sections. **d** AP score. **e** Serum amylase level. Data are presented as mean ± SEM. *n* = 4–6 mice/group. *p < 0.05, **p < 0.01, ***p < 0.001, ****p < 0.0001

An aging AAP mouse model was developed by combined treatment of EtOH feeding and CAE injections and compared to young mice. Young mice gained body weight while aging mice maintained body weight (Fig. 1a). Blood EtOH level at the experiment endpoint was higher in both young and aging mice in EtOH groups compared to the respective CON groups (*p* < 0.05, Fig. 1b). Pancreatic morphologies were apparently normal in young and aging mice fed with either diet alone, while CAE alone or combined EtOH and CAE induced inflammation (Fig. 1c). As demonstrated in Fig. 1d and e, in young mice, EtOH alone increased amylase compared to CON (*p* < 0.05); CAE alone resulted in an increased AP score and amylase (*p* < 0.05); and combined EtOH and CAE treatment had similar AP score and amylase as CAE alone. In aging mice, EtOH feeding alone did not increase AP score or amylase compared to CON; CAE alone resulted in an increased AP score (*p* < 0.05); combined EtOH and CAE treatment had 2.1-fold increased AP score and 2.6-fold increased amylase than CAE alone (*p* < 0.05). In comparison, combined EtOH and CAE treatment induced more severe AAP with 5.0-fold increased AP score and 2.4-fold increased amylase in aging mice compared to young mice. In addition, aging mice had a higher baseline AP score and amylase secretion in CON alone compared to young cohorts (*p* < 0.05). These findings suggest that EtOH and CAE have a synergistic effect on AAP severity in aging mice.

### Visium spatial transcriptomics identified distinct pancreatic tissue clusters in AAP

**Fig. 2:**
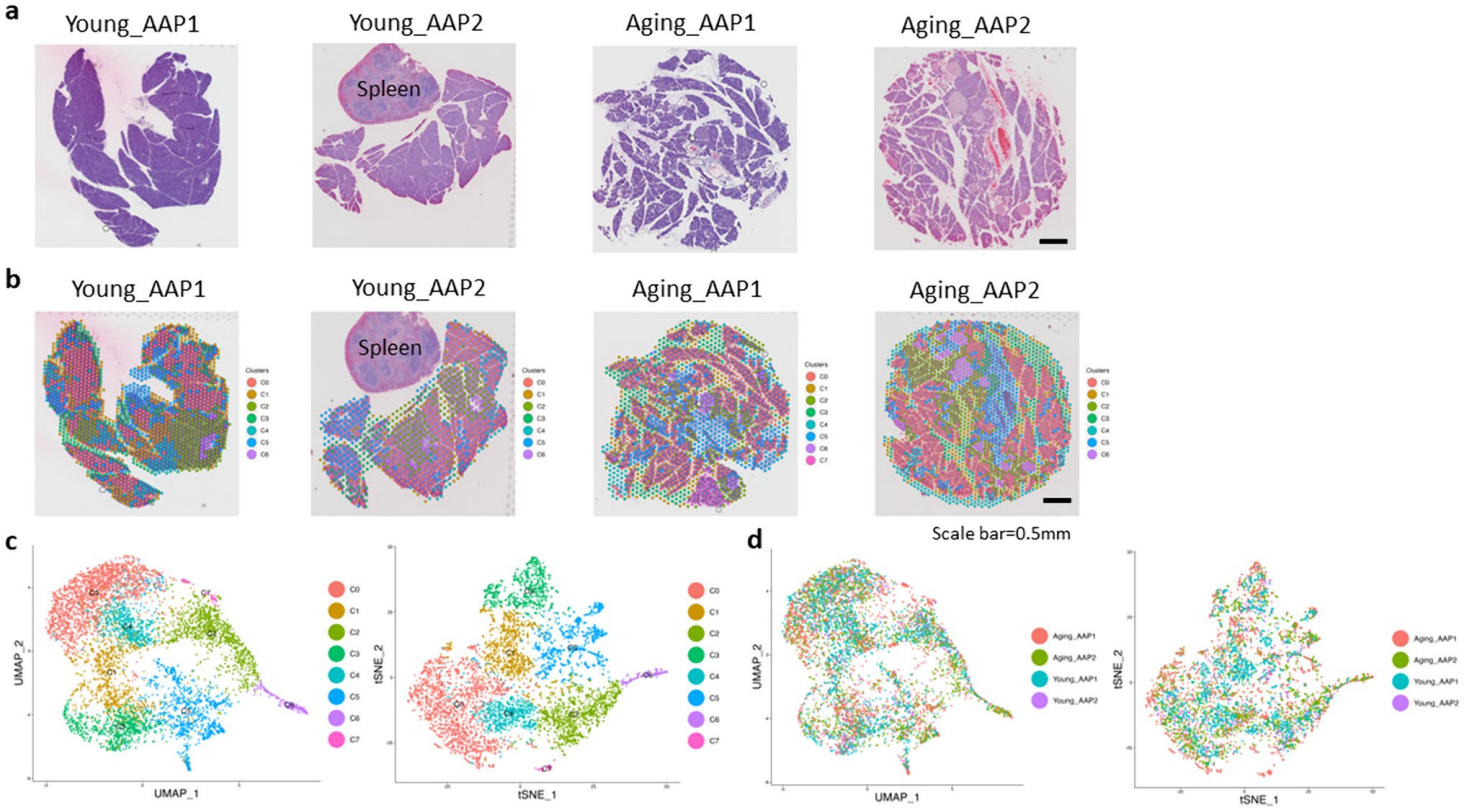
Visium spatial transcriptomics identifies distinct pancreatic clusters in AAP.** a** H&E images.** b** Clustering overlay on H&E images. **c**, **d** UMAP and t-SNE plots illustrating the annotated tissue clusters and tissue sample information within the integrated spatial transcriptomics data. The spleen in Young_AAP2 was excluded from sequencing data analysis; *n* = 2 mice/group

Visium spatial transcriptomics was performed on FFPE pancreatic tissue sections from young (Young_AAP) and aging (Aging_AAP) mice that underwent combined EtOH and CAE treatment (*n* = 2 mice/group). Transcriptomes were integrated from Young_AAP and Aging_AAP and eight distinct tissue clusters were identified and superimposed on the H&E images for visualization: clusters (C)0–6 were common to all samples; C7 was present in only one Aging_AAP sample (Fig. 2a and b). UMAP and t-SNE analyses revealed distinct, localized cluster annotations (Fig. 2c) and sample annotations (Fig. 2d) in the integrated spatial transcriptomics data.

**Fig. 3:**
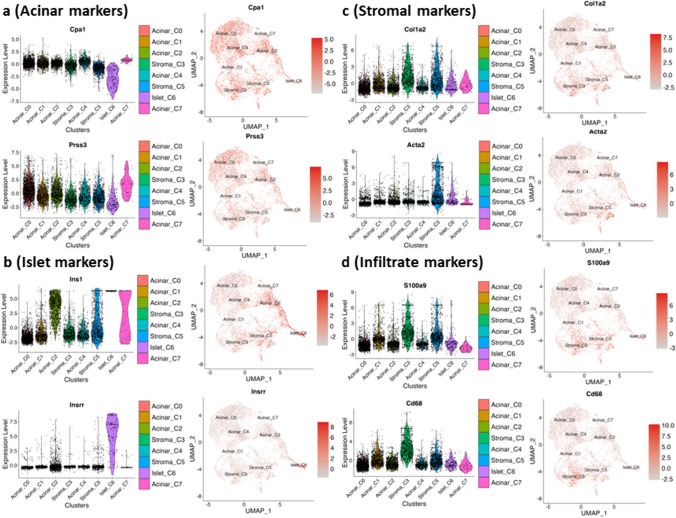
Major pancreatic tissue types are identified in each cluster.** a**–**d** Violin plot (left panel) and UMAP (right panel) showing specific gene expression levels and distribution patterns

Clusters were classified into major tissue types based on presence of cell markers identified in literature [14] and our current study (Supplemental Table 1). High expression of acinar-specific markers, *Cpa1* and *Prss3*, in C0, C1, C2, C4, and C7 suggested that these clusters mainly consisted of acinar tissue (Fig. 3a). Islet markers, *Ins1* and *Insrr*, showed that C6 consisted exclusively of islets and that C2, C5, and C7 had lower amounts of islet tissue (Fig. 3b). Stromal marker *Col1a2* revealed that C3 and C5 largely consisted of stromal tissue. Additionally, *Acta2*, a marker of myofibroblasts, was present in C5, but not in C3 (Fig. 3c). Neutrophil marker *S100a9* and macrophage marker *Cd68* revealed that C3 had neutrophil and macrophage infiltration, but C5 had mainly neutrophil infiltration (Fig. 3d). Acinar C0 covers the majority of acinar tissue. Acinar C1 had relatively high S100A9 and Cd68 levels and were in close proximity with Stromal C3 and C5; Acinar C2 and C7 have relatively high Ins1 level and were in close proximity with Islet C6, indicating that the acinar tissue is mixed with other tissue types or contains potential location-specific gene expression.

**Fig. 4:**
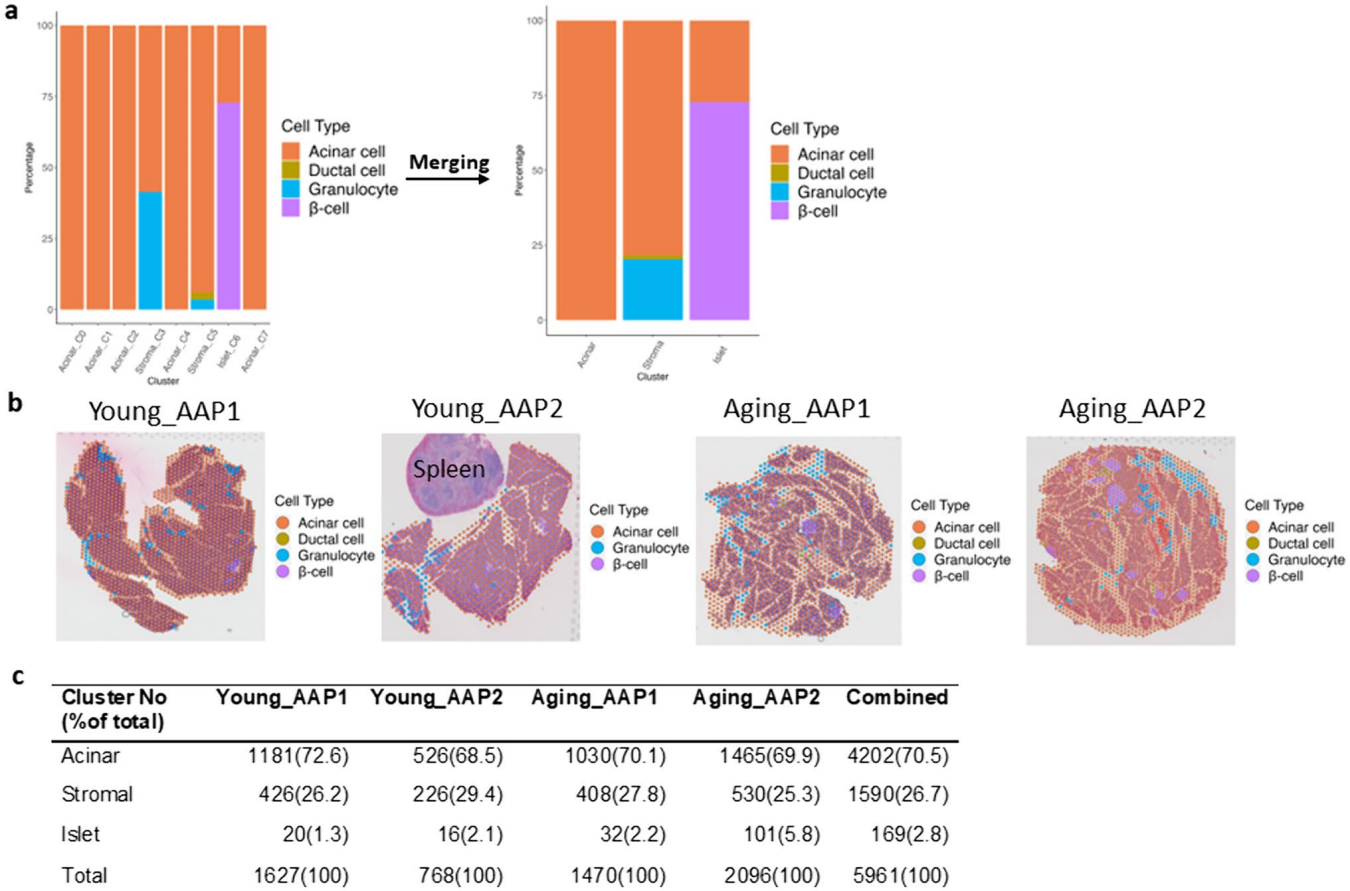
Major pancreatic tissue types in each cluster are validated by scRNA-seq integration. **a** Bar plot showing major cell types in each cluster identified with scRNA-seq data before and after cluster data merging. **b** Spatial figure plot visualizing major cell types in each sample. **c** Cluster composition

scRNA-seq data from a normal mouse pancreas [31] were used for integration analysis and further validation of the cell types present in each spatial spot. Acinar cells were predominant in C0, C1, C2, C4, and C7; granulocytes were identified in C3 and C5; ductal cells were identified in C5; and β-cells were detected in C6 (Fig. 4a). Thus, the major cell types identified by single-cell integration validated the earlier tissue marker analysis. Furthermore, based on the composition of the tissue types, data were merged into 3 main clusters, acinar, stromal, and islet. The spatial distribution of cell types was visually represented on individual pancreatic tissue samples (Fig. 4b). The compositional fraction of clusters between Young_AAP and Aging_AAP were similar except for a higher islet composition in Aging_AAP (Fig. 4c).

## Cluster-specific DEGs were predominantly upregulated in Aging_AAP

**Fig. 5:**
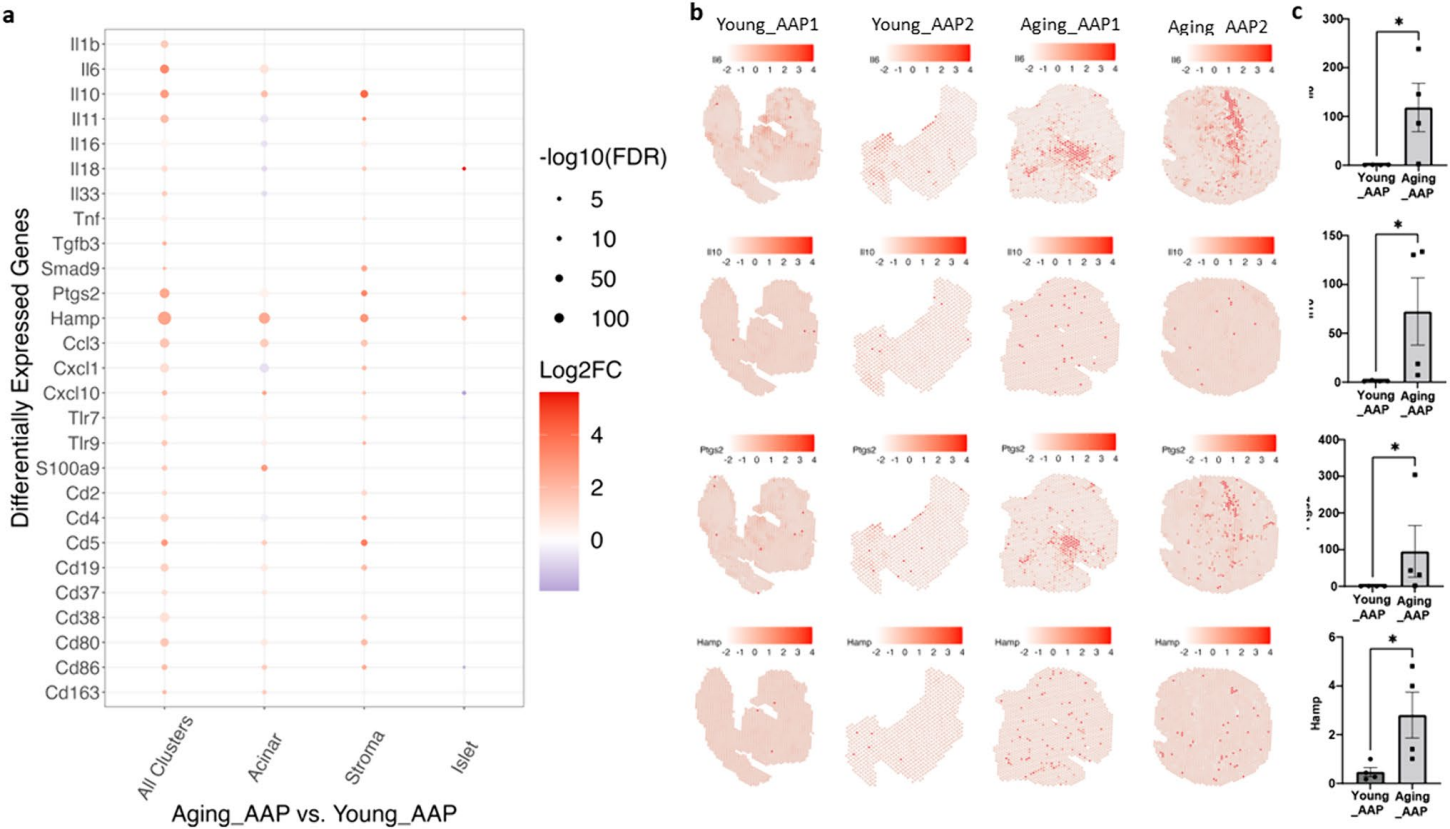
Inflammation signature genes are upregulated in Aging_AAP compared with Young_AAP.** a** Dot-plot of the inflammatory DEGs in Aging_AAP compared to Young_AAP. **b** Spatial figure plot depicting the gene expression. **c** qPCR validation. *n* = 4 mice/group. *p < 0.05

DEGs for each cluster were identified in Aging_AAP vs. Young_AAP with > 1300 DEGs in All Clusters (Supplemental Table 2). A selection of 27 DEGs were characterized for cluster-specific expression (Fig. 5a), including cytokines, transforming growth factor (TGF)-β signaling, chemokines, toll-like receptors (TLR), and cluster of differentiation (Cd) antigens [35–39]. All 27 DEGs were elevated in Aging_AAP compared to Young_AAP in all clusters (compiled DEGs from all clusters). When considering each cluster individually, more nuanced trends appeared. For instance, *Il6* was predominantly high in the acinar cluster, and *Il10* was predominantly high in acinar and stromal clusters. Paradoxical expression of several DEGs was also observed; *Il11* was high in the stromal cluster but low in the acinar cluster; *Il18* was high in stromal and islet clusters but low in the acinar cluster. A similar paradoxical expression was also observed among other DEGs. Additionally, *Hamp*, a gene that encodes hepcidin, was increased in all three clusters.

To further investigate the unique spatial expression of the key inflammatory genes, *Il6*, *Il10*, *Ptgs2*, and *Hamp* were selected for detailed analysis (Fig. 5b). Compared to Young_AAP, *Il6* was elevated in acinar cluster, *Il10* was elevated in acinar and stromal clusters, and *Ptgs2* (cyclooxygenase 2) was elevated in Aging_AAP in stromal cluster, indicating that inflammatory processes involving these genes are differentially elevated in the pancreatic tissue. *Hamp* was elevated cross all clusters. These DEG expression levels were validated with qPCR (Fig. 5c) using bulk RNA preparations from the same sets of samples used for Visium spatial transcriptomics (2 Young_AAP and 2 Aging_AAP) and additional sets of samples (2 Young_AAP and 2 Aging_AAP), presented as the combined EtOH and CAE treatment groups in Fig. 1. The top ten DEGs for All Clusters and individual clusters included genes mostly involved in inflammatory processes (Table 1). Among these, *Mmp8* and *Cd177* appeared most frequently, in all clusters and acinar and stromal clusters. Additionally, *Ppbp* and *Hamp* appeared in all clusters and acinar cluster.

**Table 1:** Top 10 DEGs for each cluster in Aging_AAP compared to Young_AAP

	**All**^**b**^		**Acinar**		**Stroma**		**Islet**	
**Rank**^**a**^	**Gene**	**Log2FC**	**Gene**	**Log2FC**	**Gene**	**Log2FC**	**Gene**	**Log2FC**
1	Mmp8	2.24	Ppbp	1.31	Sult1e1	3.14	Ctse	2.82
2	Ppbp	2.17	Cxcr2	0.97	Fcrlb	3.93	Spef2	^c^0.26
3	Serpina3m	2.98	Mmp8	1.80	Pdcd1	1.78	Pmel	5.89
4	Cxcl13	2.59	Hamp	2.38	Gpr84	5.39	Mybpc2	5.22
5	Hamp	2.52	Cd177	1.40	Ankrd66	2.18	5730522E02Rik	4.44
6	Cd177	1.98	Ivl	1.81	Nfe2	2.15	Il18r1	5.93
7	Fga	3.15	Nxph3	1.12	Trdj2	3.90	Nek11	4.25
8	Mmp25	1.48	Gpat3	1.69	Ptprh	3.34	Ighj4	2.46
9	Tarm1	1.10	Wfdc21	1.34	Mmp8	2.67	Cmya5	3.53
10	Acod1	1.88	Mmp24	1.21	Cd177	2.93	Rasl11b	3.02

^a^Ranked according to FDR (< 0.05)

^b^Compiled from all clusters

^c^Downregulated genes

## Cluster-specific differential pathways relevant to immune responses were regulated in Aging_AAP

**Fig. 6:**
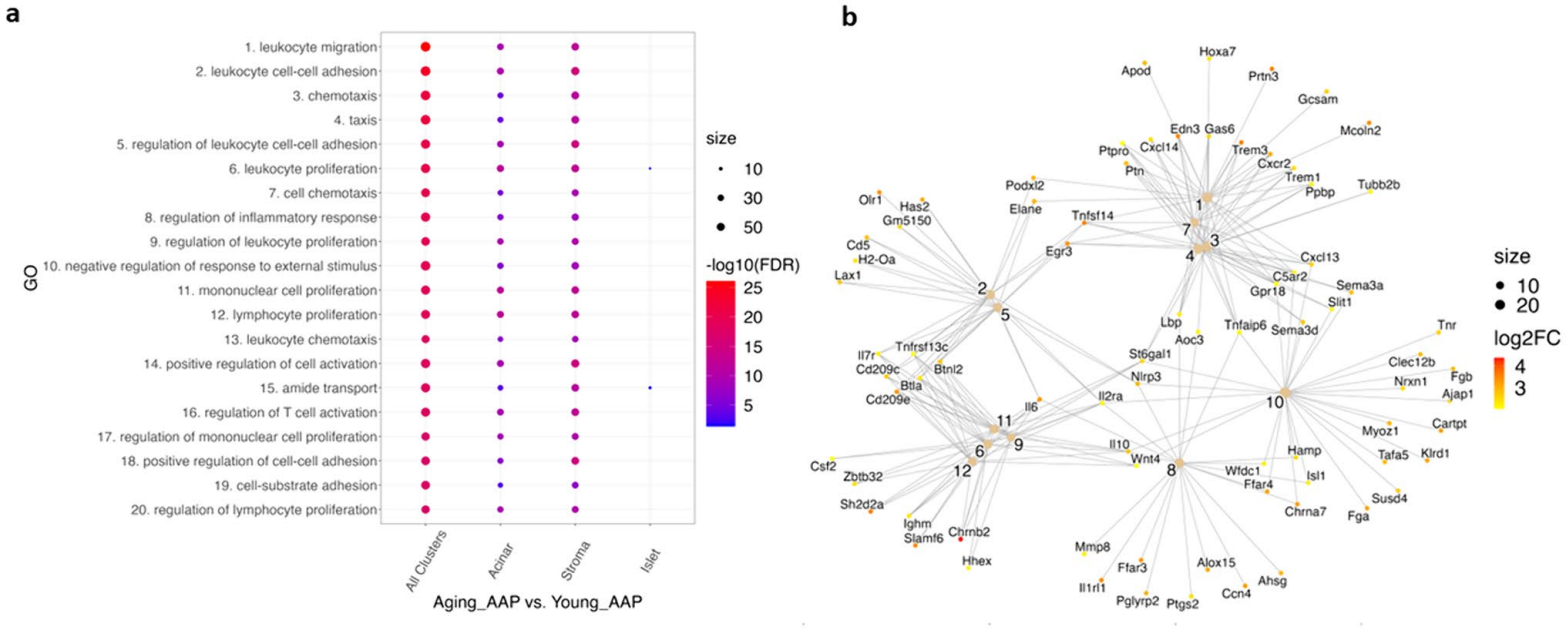
Differential pathways are upregulated in Aging_AAP compared with Young_AAP. **a** Dot plot of the top 20 differential pathways in Aging_AAP compared to Young_AAP. **b** Interaction plot of the top 12 differential pathways from all clusters. Size: number of DEGs

To investigate the cluster-specific functional differences between Aging_AAP and Young_AAP, ORA of GO was applied, and around 3000 differential pathways were derived from the aggregated DEGs in all clusters (Supplemental Table 3). Compared to Young_AAP, the top 20 upregulated pathways in Aging_AAP were mainly involved in immune responses (Fig. 6a). Cluster-specific analysis showed tissue-dependent trends. The stromal tissue has relatively increased inflammatory pathways mostly on leukocyte migration, adhesion, chemotaxis, and proliferation. Notably, only 2 pathways, leukocyte proliferation, and amide transport, out of the top 20 differential pathways appeared in the islet cluster. This might be attributed to a lack of strong inflammatory response in islet tissue.

DEGs involved in the top 12 differential pathways from all clusters were directly compared (Fig. 6b). Many of these differential pathways have an appreciable overlap of genes. Pathways 1, 3, 4, and 7, which are involved in leukocyte migration and chemotaxis, include common DEGs such as *Trem3*, *Cxcr2*, *Trem1*, *Ppbp*, *Cxcl13*, *Tnfaip6*, *Lbp*, *Tnfsf14*, *Ptn*, *Ptpro*, and *Gas6*. Pathways 6, 9, 11, and 12 involve leukocyte proliferation and include common DEGs such as *Chrnb2*, *Ighm*, *Cd209e*, *Btnl2*, *Tnfrsf13c*, *Il6*, *Il2ra*, and *Il10*. Pathways 2 and 5 involve leukocyte adhesion and share several common DEGs including *Tnfsf14*, *Erg3*, *Nlrp3*, *Il2ra*, *Il6*, *Il7r*, *Lax1*, *H2-Oa*, *Cd5*, *Gm5150*, *Has2*, and *Elane*. Pathways 8 and 10 involve inflammatory response and negative regulation of response to external stimulus respectively, and include common DEGs such as *Chrna7*, *Ffar4*, *Wfdc1*, *Isl1*, *Hamp*, *Il10*, *Il2ra*, *Nlrp3*, and *Tnfaip6*. The intersections of these pathways exemplify the role of DEGs and their contribution to the increased severity of inflammation observed in Aging_AAP.

Taken together, the main differences of DEGs and regulated pathways fall onto the acinar and stromal clusters with fewer changes in the Islet cluster, suggesting that the acinar and stromal tissues are more likely impacted by AAP than the Islet tissue.

## Discussion

Alcohol is suggested to exacerbate disease in older adults [40, 41]. To explore the underlying mechanisms of alcohol and aging on AP, we established an aging AAP mouse model and found that aging mice had significantly worse AAP than young mice, suggesting that alcohol feeding has a marked synergistic effect on disease severity when combined with caerulein in aging mice. We then applied spatial transcriptomics to both aging and young AAP mouse pancreatic tissue sections in order to understand spatial molecular alterations and mechanisms in the heterogenous tissue of the pancreas with AAP.

The fate of acinar cells plays an important role in clinical outcomes in AP. Acinar cells are involved in the initiation of AP and undergo apoptosis or necrosis, releasing cytotoxic remnants and leading to increased disease severity [42]. Acinar cells are reported to be a major source of inflammatory cytokines, which can be exacerbated by alcohol consumption [43]. Our study deepened the understanding of the role of acinar cells.

The DEG expression profiles and differential pathways relevant to inflammation in Aging_AAP varied widely in the acinar cluster with some DEGs being elevated and some DEGs being downregulated in Aging_AAP compared to Young_AAP (Supplemental Table 2), indicating heterogeneous inflammation patterns in acinar tissue during AAP. Contrastingly, the stromal cluster had the most prominent inflammation, with a majority of DEGs being elevated in Aging_AAP compared to Young_AAP. This pattern was paralleled in the analysis of pathways (Supplemental Table 3), which showed a majority of differential pathways elevated in the stromal cluster when comparing Aging_AAP and Young_AAP. This spatial mapping suggests that the inflammation is greatest at the stromal tissue of the pancreas during AAP.

This study has several aspects of novelty. Our established aging AAP mouse model in this study recaptured the effects of aging and alcohol consumption in humans with AP, providing a clinically relevant and feasible model for mechanistic studies regarding increased sensitivity of aging population to the deleterious effects of alcohol consumption in AAP. This study is the first reported utilization of spatial transcriptomics in AAP leading to the discovery of extensive contrast between aging and young AAP. The effective utilization of FFPE sections for spatial transcriptomics can be extended to clinically archived human FFPE samples. However, this study has several limitations. First, we did not include the caerulein control in the spatial transcriptomics. Thus, as a caution for interpretation of the spatial transcriptomic data, our current data analysis discovered AAP-specific DEGs, which may be compounded with caerulein (or AP)-induced DEGs. However, our study may also provide additional information on both AP- and AAP-relevant gene regulation. The resolution of spatial transcriptomics is not at the single-cell level, as each spatial spot represents 1–10 cells, and Seurat, used in this study for integration analysis, can only identify the dominant cell type in each spatial spot. Future studies on generating scRNA-seq data or staining for specific cell types could provide valuable insights on the inflammatory responses at the single cell level on this line of research. Male mice were used in this experiment and further studies are necessary to determine the sex-based effects. The sample size (*n* = 2 mice/group) for the Visium spatial transcriptomics was small but was selected from the larger groups (*n* = 4–6 mice/group) with validated functional outcomes. Visium spatial transcriptomics was only performed at a single time point following AAP induction. As a proof-of-concept study, we identified the main differences of pancreatic spatial transcriptomes between Aging_AAP and Young_AAP and demonstrated feasibility for future detailed studies regarding the molecular mechanisms and potential drivers for AAP.

In conclusion, our study established a clinically relevant aging AAP mouse model and applied Visium spatial transcriptomics to the pancreatic tissue sections collected from this model. Our work revealed the spatial organization of the transcriptome in functionally specialized clusters of the inflamed pancreatic tissues in AAP state, demonstrated spatial heterogeneity of inflammatory processes in AAP mice, and uncovered extensive differences between aging and young AAP. Further investigation of the molecular mechanisms may provide novel insights that can lead to targeted therapeutics and improved outcomes for aging AAP.

### Supplementary Information

Below is the link to the electronic supplementary material.

Supplementary file1 (XLSX 315 KB)

Supplementary file2 (XLSX 248 KB)

Supplementary file3 (XLSX 648 KB)

## Data Availability

Spatial transcriptomic data have been deposited at GEO (GSE235247). Any additional information reported in this paper is available from the corresponding author on reasonable request.

## Abbreviations

AP: Acute pancreatitis
AAP: Alcohol-induced acute pancreatitis
EtOH: Alcohol
CAE: Caerulein
DEGs: Differentially expressed genes
